# Correlation between dietary selenium intake and stroke in the National Health and Nutrition Examination Survey 2003–2018

**DOI:** 10.1080/07853890.2022.2058079

**Published:** 2022-05-20

**Authors:** Wenrui Shi, Liang Su, Jian Wang, Fangze Wang, Xu Liu, Jianxin Dou

**Affiliations:** a Department of Cardiology, Shanghai Chest Hospital, Shanghai Jiao Tong University, Shanghai, China; b Department of Cardiology II, Weifang People’s Hospital, Weifang, China; c Department of Endocrinology, Weifang People’s Hospital, Weifang, China

**Keywords:** Dietary selenium intake, stroke, negative correlation, non-linear model, adults, National Health And Nutrition Examination Survey

## Abstract

**Background:**

Epidemiologic evidence of the effect of dietary selenium intake on stroke risk remains controversial. This study aimed to examine the cross-sectional correlation between dietary selenium intake and the risk of stroke in adults.

**Materials and methods:**

We retrospectively analysed 39,438 participants from the National Health and Nutrition Examination Survey 2003–2018, aged 20–85 years. Participants were divided into quartiles depending on daily dietary selenium intake: quartile 1 (0–77 μg), quartile 2 (77–108 μg), quartile 3 (108–148 μg), and quartile 4 (148–400 μg). The dose-response relationship was assessed using the restricted cubic spline function.

**Results:**

The adjusted odds ratios (ORs) and 95% confidence intervals (CIs) of stroke were 0.70 (0.55, 0.88) for participants in quartile 2, 0.71 (0.53, 0.93) for quartile 3, and 0.61 (0.43, 0.85) for quartile 4 compared with that in quartile 1. *p*-Value for trend through quartiles was .007. A non-linear negative correlation between dietary selenium intake and stroke was observed in the threshold effect analysis and restricted cubic spline function (*p*-value for non-linearity < .001). An initial decrease in odds of stroke lower than 105 μg/day selenium intake (0.61 [0.44, 0.85], *p* = .004) was followed by a platform beyond 105 μg/day (0.97 [0.81, 1.16], *p* = .723). In the subgroup analysis, adjusted ORs (95% CIs) of stroke were 0.51 (0.36, 0.70) for female participants, 0.63 (0.40, 0.99) for participants with age <60 years, 0.63 (0.47, 0.85) for participants with poverty-income ratio < 2.14, 0.66 (0.50, 0.87) for participants with overweight and obesity, 0.66 (0.52, 0.84) for participants with hypertension, 0.72 (0.53, 0.97) for participants without diabetes, and 0.72 (0.56, 0.92) for non-anaemic participants.

**Conclusions:**

Dietary selenium had a negative and non-linear correlation with the risk of stroke in adults. The correlation varied across different population subgroups.

**KEY MESSAGES**
- Dietary selenium had a negative and non-linear correlation with the risk of stroke in adults.
- Non-linear negative correlation trends were observed in subpopulations of females, age <60 years, poverty-income ratio <2.14, overweight and obesity, hypertension, non-diabetes, and non-anaemia.
- Dietary selenium intake of approximately 105 μg per day has an optimum effect on stroke.

## Introduction

Stroke is a significant cause of mortality and disability worldwide [1,2]. Millions of Americans experience a new or recurrent stroke every year, which can lead to long-term disability [1,2]. The global lifetime risk of stroke has increased from 22% to 24% over the past three decades [3]. With the ageing population, the burden of stroke continues to increase, especially in developing countries [3]. There are limited treatment options for patients that have undergone a stroke. Hence, there remains an urgent need to identify novel strategies for stroke prevention. Selenium, as an essential trace element, has recently attracted significant attention because of its beneficial effects on stroke risk [4,5].

Selenium, as selenocysteine, is a crucial component of selenoproteins, a class of proteins primarily involved in anti-oxidation and redox regulation [6,7]. Oxidative stress has recently been considered a critical pathophysiological mechanism in stroke [8]. Previous observational studies have reported the beneficial effects of selenium on cardiovascular diseases, including stroke [9,10]. However, secondary analyses in the Nutritional Prevention of Cancer (NPC) trial did not reveal any benefit of selenium supplementation on the risk of stroke [9,10].

Notably, epidemiological studies have mainly focussed on the benefits of circulating selenium levels but not on selenium intake. In the NPC trial, we believe selenium supplementation (200 μg/day) could be slightly high for stroke prevention [11]. Following the hypothesis that selenium supplementation has a threshold effect or “U” shape effect on the risk of stroke, we examined the dose-response correlation between dietary selenium intake and the risk of stroke in the National Health and Nutrition Examination Survey (NHANES) 2003–2018.

## Materials and methods

### Study population

The NHANES is a cross-sectional investigation that provides comprehensive information about the nutrition and health of the national population in the United States [12]. Data from eight consecutive NHANES with 2-year cycles (2003–2004, 2005–2006, 2007–2008, 2009–2010, 2011–2012, 2013–2014, 2015–2016, 2017–2018) were collected. All participants provided informed consent, and the ethics approval was obtained from the research ethics review board at the National Centre for Health Statistics [13].

### Exposure and outcomes

Dietary selenium intake from foods was calculated using the US Department of Agriculture's Food and Nutrient Database for Dietary Studies. Two non-consecutive days of intake data were available for each participant during 2003–2018. The first day’s data was collected at a mobile examination centre, and the second day’s data was collected over telephone 3–10 days later. Selenium intake from supplements reflects average daily selenium intake from non-prescription and prescription dietary supplements during the 30-day period before the survey date. In this analysis, the average selenium intake from foods and supplements was added together as dietary selenium intake. The tolerable upper level of selenium intake for adults is 400 μg [14]. Therefore, the upper limit of daily selenium intake was set at 400 μg in this study.

A questionnaire was used to record whether the patients had pre-existing medical conditions. Stroke was defined using self-reported history by asking the following question: "Has a doctor ever told you that you had a stroke?" The exclusion criteria were as follows: (1) individuals who refused to answer this question; (2) individuals who did not know if they ever had a stroke.

### Covariates

Variables of interest obtained by questionnaires included basic information of participants on age (years), sex (male, %), race, education level, marital status (married, %), hypertension (self-reported), diabetes (self-reported), family poverty-income ratio (PIR), body mass index (BMI), smoking (smoked at least 100 cigarettes during their lifetime or not), alcohol use (consumed at least 12 alcoholic drinks per year or not), and physical activity (never, moderate, and vigorous). According to the analysis of previous related studies [15,16], the following covariates were included: the levels of total cholesterol, high-density lipoprotein-cholesterol (HDL-cholesterol), triglycerides, glycohemoglobin, and the daily intake of total energy and cholesterol from the diet. Haemoglobin and uric acid levels were included because of anaemia and hyperuricaemia association with stroke [17,18].

The PIR was calculated as family income divided by the poverty threshold specific to family size and the appropriate year and state. The patients were divided into two groups with a median of 2.14 for subgroup analysis. BMI was calculated as body weight divided by height squared, and the participants were categorised as normal weight (<25.0 kg/m^2^), overweight (25.0–29.9 kg/m^2^) and obesity (≥30.0 kg/m^2^) [19]. Vigorous physical activity was defined as activity that significantly increased breathing or heart rate, whereas moderate physical activity was defined as activity that slightly increased breathing rate. Haemoglobin levels were categorised into non-anaemia (≥12 g/dL) and anaemia (<12 g/dL).

### Statistical analysis

The recommended sample weights were used in all analyses. The recommended 2-year sample weight for the 2003–2018 period was used to calculate the new 16-year sample weights for all participants.

Continuous variables were expressed as mean ± standard deviation (SD), and categorical variables were expressed as numbers (percentage). Odds ratios (ORs) and 95% confidence intervals (95% CIs) of stroke were estimated with EmpowerStats software using multivariate logistic regression and a piece-wise linear regression model. Three adjusted models were developed: Model 1 was adjusted for age, sex, and race. Model 2 was adjusted for education level, marital status, PIR, BMI, smoking, alcohol use, hypertension, diabetes, and physical activity based on Model 1. Model 3 was adjusted for levels of haemoglobin, uric acid, total cholesterol, HDL-cholesterol, triglyceride, glycohemoglobin, daily intake of total energy, and cholesterol from the diet based on Model 2. The dose-response relationship between dietary selenium intake and stroke was assessed using Stata version 15.0, using a restricted cubic spline function with four knots located at the 25th, 50th, 75th, and 99th percentiles, and *p*-value for non-linearity was calculated by testing the null hypothesis that the coefficient of the second spline is equal to 0 [20]. Statistical significance was set at *p* < .05.

## Results

### Clinical characteristics of included participants

A total of 44,790 participants (aged 20–85 years) responded to the question, “Has a doctor ever told you that you had a stroke?” After excluding 68 participants who refused to answer this question or did not know whether they had a stroke, 941 participants who were pregnant, 4200 participants without information on dietary selenium intake, and 143 participants whose dietary selenium intake >400 μg/day, 39,438 participants were finally included in this study. Among the 39,438 participants, there are 38,695 participants providing information on selenium intake from foods and 8635 participants providing information on selenium intake from supplements. The characteristics of the included participants are shown as quartiles in Table 1.

**Table 1.** Description of participants included in the present study.

Quartiles of dietary selenium intake (100 μg/day)	Quartile1 (0.00–0.77)	Quartile2 (0.77–1.08)	Quartile3 (1.08–1.48)	Quartile4 (1.48–4.00)	*p*-Value
Case number	9858	9861	9855	9864	
Incidence of stroke	584 (5.92%)	411 (4.17%)	339 (3.44%)	256 (2.60%)	<.001
Age, years	53.07 ± 18.63	50.93 ± 18.14	49.28 ± 17.63	47.63 ± 16.95	<.001
Male, *n* (%)	3014 (30.57%)	4086 (41.44%)	5281 (53.59%)	7078 (71.76%)	<.001
Race, *n* (%)					<.001
Mexican American	1583 (16.06%)	1624 (16.47%)	1583 (16.06%)	1472 (14.92%)	
Other Hispanic	921 (9.34%)	860 (8.72%)	875 (8.88%)	817 (8.28%)	
Non-Hispanic White	4045 (41.03%)	4367 (44.29%)	4420 (44.85%)	4482 (45.44%)	
Non-Hispanic Black	2455 (24.90%)	2106 (21.36%)	1951 (19.80%)	1929 (19.56%)	
Other Race	854 (8.66%)	904 (9.17%)	1026 (10.41%)	1164 (11.80%)	
Education level, *n* (%)					<.001
Less than 9th grade	1549 (15.74%)	1155 (11.72%)	931 (9.46%)	649 (6.58%)	
9–11th grade	1603 (16.28%)	1411 (14.32%)	1342 (13.63%)	1227 (12.45%)	
High school graduate	2414 (24.52%)	2313 (23.48%)	2210 (22.45%)	2248 (22.81%)	
Some college or AA degree	2661 (27.03%)	2882 (29.25%)	2970 (30.17%)	3017 (30.61%)	
College graduate or above	1617 (16.43%)	2091 (21.22%)	2392 (24.30%)	2716 (27.55%)	
Married, *n* (%)	4608 (46.78%)	4971 (50.43%)	5335 (54.17%)	5402 (54.79%)	<.001
Alcohol use, *n* (%)	2359 (23.93%)	2344 (23.77%)	2093 (21.24%)	1954 (19.81%)	<.001
Smoking, *n* (%)	4355 (44.18%)	4372 (44.34%)	4496 (45.62%)	4704 (47.69%)	<.001
Diabetes, *n* (%)	1465 (14.86%)	1321 (13.40%)	1227 (12.45%)	1101 (11.16%)	<.001
Hypertension, *n* (%)	3990 (40.47%)	3662 (37.14%)	3427 (34.77%)	3330 (33.76%)	<.001
Physical activity*, n* (%)					<.001
Never	4215 (43.34%)	3608 (36.87%)	3153 (32.15%)	2646 (26.88%)	
Moderate	3018 (31.03%)	3141 (32.10%)	3088 (31.49%)	2817 (28.62%)	
Vigorous	2493 (25.63%)	3037 (31.03%)	3566 (36.36%)	4379 (44.49%)	
Poverty-income ratio	2.24 ± 1.54	2.49 ± 1.60	2.62 ± 1.63	2.78 ± 1.65	<.001
Body mass index, kg/m^2^	29.15 ± 7.02	29.17 ± 6.84	29.27 ± 6.97	28.90 ± 6.74	.002
Haemoglobin, g/dL	13.71 ± 1.52	13.96 ± 1.52	14.21 ± 1.52	14.55 ± 1.46	<.001
Uric acid, mg/dL	5.32 ± 1.48	5.38 ± 1.43	5.51 ± 1.42	5.67 ± 1.39	<.001
Total cholesterol, mg/dL	195.01 ± 42.43	194.44 ± 42.28	193.74 ± 41.46	192.16 ± 41.98	<.001
HDL-cholesterol, mg/dL	54.31 ± 16.59	53.73 ± 16.15	52.54 ± 15.90	51.49 ± 15.60	<.001
Triglyceride, mg/dL	144.29 ± 108.55	148.01 ± 122.15	157.49 ± 141.16	160.48 ± 155.04	<.001
Glycohemoglobin, %	5.80 ± 1.10	5.76 ± 1.07	5.76 ± 1.09	5.72 ± 1.04	<.001
Blood selenium (ng/mL)	177.59 ± 34.78	180.62 ± 32.39	185.60 ± 30.91	192.28 ± 33.48	<.001
Dietary intake per day					
Total energy (kcal)	1337.85 ± 469.55	1791.64 ± 501.70	2176.21 ± 622.98	2771.57 ± 934.23	<.001
Cholesterol (mg)	148.84 ± 91.73	240.99 ± 124.00	316.41 ± 160.27	439.91 ± 238.74	<.001
Selenium (100 μg)	0.55 ± 0.16	0.92 ± 0.09	1.26 ± 0.12	2.01 ± 0.49	<.001

Continuous variables were described using mean ± standard deviation (SD) and were analysed by the *t*-test. Categorical variables were expressed as numbers (percentage) and were analysed by the chi-square test.

### Correlation between dietary selenium intake and risk of stroke

As shown in Table 2, when dietary selenium intake was assessed as quartiles after multivariate adjustment for all the above-mentioned covariates in Model 3, the OR (95% CI) of stroke was 0.70 (0.55, 0.88) for participants in quartile 2 (77–108 μg/day), 0.71 (0.53, 0.93) for quartile 3 (108–148 μg/day), and 0.61 (0.43, 0.85) for quartile 4 (148–400 μg/day) compared with that in quartile 1 (0–77 μg/day). The *p*-value for trend through quartiles was .007.

**Table 2.** Adjusted odds ratios of stroke correlated with dietary selenium intake.

Dietary selenium intake (100 μg/day, *n* = 39,438)	Model 1	Model 2	Model 3
Quartile 1 (0.00–0.78)	Ref.	Ref.	Ref.
Quartile 2 (0.78–1.08)	0.65 (0.54, 0.78)	0.70 (0.56, 0.86)	0.70 (0.55, 0.88)
*p*-Value	<.001	.001	.004
Quartile 3 (1.08–1.48)	0.62 (0.49, 0.77)	0.72 (0.56, 0.91)	0.71 (0.53, 0.93)
*p*-Value	<.001	.009	.017
Quartile 4 (1.48–4.00)	0.48 (0.39, 0.60)	0.62 (0.48, 0.80)	0.61 (0.43, 0.85)
*p*-Value	<.001	<.001	.005
*p*-Value for trend	<.001	<.001	.007

Model 1 was adjusted for age, sex, and race. Model 2 was adjusted for education level, marital status, poverty-income ratio, body mass index, smoking, alcohol use, hypertension, diabetes, physical activity based on Model 1. Model 3 was adjusted for levels of haemoglobin, uric acid, total cholesterol, HDL-cholesterol, triglyceride, glycohemoglobin, daily intake of total energy and cholesterol from the diet based on Model 2.

### Threshold effect of dietary selenium intake on risk of stroke

**Figure 1.**
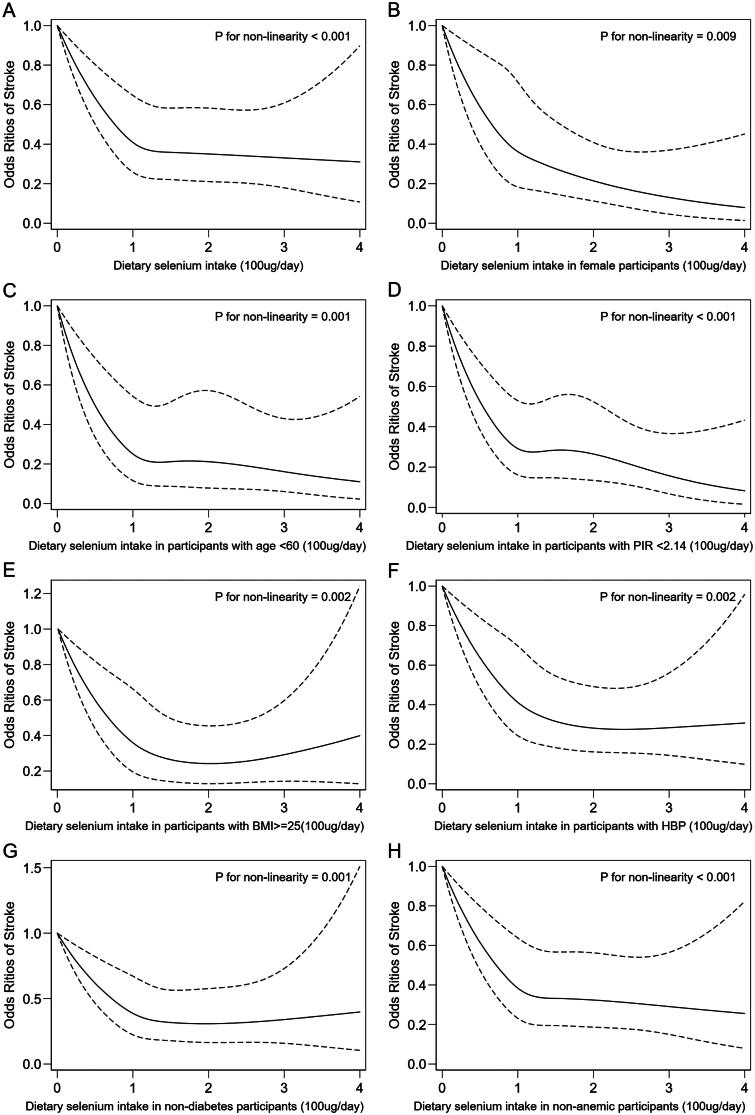
The weighted odds ratio of stroke correlated with dietary selenium intake. (A) All participants. (B) Female participants. (C) Participants with age <60 years. (D) Participants with PIR <2.14. (E) Participants with overweight and obesity. (F) Participants with hypertension. (G) Participants without diabetes. (H) Participants without anaemia. The solid and long dash lines represent the estimated odds ratio and 95% confidence interval. Odds ratios were adjusted for age, sex, race, education level, marital status, poverty-income ratio, body mass index, smoking, alcohol use, hypertension, diabetes, physical activity, levels of haemoglobin, uric acid, total cholesterol, HDL-cholesterol, triglyceride, glycohemoglobin, daily intake of total energy and cholesterol from the diet. PIR: family poverty-income ratio.

After multivariate adjustment for all covariates mentioned above in Model 3, a non-linear negative correlation was observed between dietary selenium intake and the risk of stroke, with an obvious breakpoint at 105 μg/day (*p*-value for non-linearity = .025). There was an initial decrease (<105 μg/day) (0.61 [0.44, 0.85], *p* = .004) in odds followed by a platform beyond 105 μg/day (0.97 [0.81, 1.16], *p* = .723) (Table 3). A non-linear negative correlation between dietary selenium intake and risk of stroke was observed by the spline smoothing plot in Figure 1(A) (*p*-value for non-linearity <.001).

**Table 3.** Threshold effect of dietary selenium intake on risk of stroke.

Dietary selenium intake (100 μg/day, *n* = 39,438)	Model 1	Model 2	Model 3
Linear model			
OR (95% CI) *p*-value	0.71 (0.64, 0.78) <.001	0.81 (0.72, 0.90) <.001	0.86 (0.74, 1.00) . 051
Non-linear model			
Breakpoint (*K*)	2.00	1.20	1.05
OR1(95% CI), <*K* *p*-value	0.63 (0.55, 0.71) <.001	0.62 (0.49, 0.77) <.001	0.61 (0.44, 0.85) . 004
OR2(95% CI), >*K* *p*-value	1.22 (0.88, 1.71) .237	0.99 (0.83, 1.18) .889	0.97 (0.81, 1.16) .723
*p*-Value for non-linearity	.002	.007	.025

The piece-wise linear regression model was applied to show the threshold effect of dietary selenium intake on the risk of stroke. Linear model: model that presumes the correlation between dietary selenium intake and the risk of stroke is linear. Non-linear model: model that presumes the correlation between dietary selenium intake and the risk of stroke is non-linear and has a breakpoint. *p*-Value for non-linearity <.05 means that the non-linear model may better describe the correlation. Model 1 was adjusted for age, sex, and race. Model 2 was adjusted for education level, marital status, poverty-income ratio, body mass index, smoking, alcohol use, hypertension, diabetes, physical activity based on Model 1. Model 3 was adjusted for levels of haemoglobin, uric acid, total cholesterol, HDL-cholesterol, triglyceride, glycohemoglobin, daily intake of total energy and cholesterol from the diet based on Model 2. OR (95% CI): odds ratio and 95% confidence interval.

### Stratified correlations between dietary selenium intake and stroke

Non-linear negative correlation trends were observed in subgroups of female, age <60, PIR <2.14, overweight and obesity, hypertension, non-diabetes, and non-anaemia (Figure 1(B–H)). Adjusted ORs (95% Cls) of stroke were 0.51 (0.36, 0.70) for female participants, 0.63 (0.40, 0.99) for participants with age <60 years, 0.63 (0.47, 0.85) for participants with PIR <2.14, 0.66 (0.50, 0.87) for participants with overweight and obesity, 0.66 (0.52, 0.84) for participants with hypertension, 0.72 (0.53, 0.97) for participants without diabetes, and 0.72 (0.56, 0.92) for non-anaemic participants (Table 4). All *p*-values for non-linearity in these subgroups <.01.

**Table 4.** Subgroups analysis for the correlation between dietary selenium intake and the risk of stroke.

Subgroups	Incidence of stroke	Levels of blood selenium (ng/mL)	Dietary selenium intake (100 μg/day)	ORs (95%CIs) of stroke	*p*-Value
Male	2.62%	186.16 ± 33.21	1.37 ± 0.65	1.00 (0.74, 1.35)	.986
Female	3.23%	182.11 ± 33.43	1.01 ± 0.49	0.51 (0.36, 0.70)	<.001
Age <60 years	1.28%	187.13 ± 30.06	1.23 ± 0.61	0.63 (0.40, 0.99)	.046
Age > =60 years	7.64%	179.22 ± 37.63	1.10 ± 0.56	0.82 (0.64, 1.06)	.127
PIR <2.14	4.36%	183.31 ± 33.35	1.13 ± 0.59	0.63 (0.47, 0.85)	.002
PIR > =2.14	2.11%	184.53 ± 33.89	1.25 ± 0.61	0.86 (0.62, 1.19)	.349
Normal weight	2.29%	183.68 ± 33.52	1.20 ± 0.61	1.03 (0.68, 1.55)	.902
Overweight/obesity	3.05%	184.54 ± 33.22	1.19 ± 0.59	0.66 (0.50, 0.87)	.004
Non-smoking	2.27%	186.17 ± 32.32	1.17 ± 0.60	0.79 (0.57, 1.10)	.168
Smoking	3.73%	181.58 ± 34.45	1.20 ± 0.61	0.72 (0.51, 1.01)	.055
Non-hypertension	1.11%	185.43 ± 32.32	1.21 ± 0.61	0.97 (0.64, 1.47)	.875
Hypertension	6.81%	182.12 ± 34.84	1.15 ± 0.59	0.66 (0.52, 0.84)	.001
Non-diabetes	2.19%	183.92 ± 33.00	1.20 ± 0.60	0.72 (0.53, 0.97)	.034
Diabetes	9.55%	184.63 ± 34.56	1.13 ± 0.58	0.77 (0.53, 1.13)	.188
Non-anaemia	2.70%	185.09 ± 33.48	1.21 ± 0.60	0.72 (0.56, 0.92)	.010
Anaemia	7.14%	172.59 ± 29.96	1.00 ± 0.49	1.02 (0.52, 2.01)	.947

Continuous variables were described by using mean ± standard deviation (SD). ORs were adjusted for age, sex, race, education level, marital status, poverty-income ratio, body mass index, smoking, alcohol use, hypertension, diabetes, physical activity, levels of haemoglobin, uric acid, total cholesterol, HDL-cholesterol, triglyceride, glycohemoglobin, daily intake of total energy and cholesterol from the diet. ORs (95% CIs): odds ratios and 95% confidence intervals; PIR: poverty-income ratio.

## Discussion

In this study, we observed a non-linear negative correlation between dietary selenium intake and the risk of stroke in adults. A significant decrease in the risk of stroke was observed until 105 μg dietary selenium was consumed per day. Previous studies have shown a correlation between selenium deficiency and cardiovascular diseases [21,22]. Therefore, selenium supplementation to improve health is becoming increasingly popular. However, selenium is toxic at high levels [23]. The recommended daily adequate selenium intake is 70 μg for adults with a tolerable upper intake level of 400 μg/day [14,24]. Our research shows that a selenium intake of approximately 105 μg per day has an optimum beneficial effect on the risk of stroke. Increasing selenium intake has no further benefit. This finding validates our previous hypothesis and may explain why selenium supplementation (200 μg/day) had no effect on stroke risk in the NPC trial.

The correlation between selenium levels and stroke may vary across different population subgroups. The decline in the risk of stroke was significant in women and participants with lower income, overweight and obesity, or hypertension, probably because of the low serum selenium concentration and low habitual selenium intake [25–27]. The incidence of stroke was high among these participants. This could be improved by appropriate selenium supplementation, suggesting that selenium deficiency may play a role in the incidence of stroke in these populations.

We observed a negative correlation between dietary selenium intake and the risk of stroke in non-anaemic individuals. In contrast, no correlation was found between dietary selenium intake and the risk of stroke in anaemic individuals despite low blood selenium levels and low selenium intake, which suggested that anaemia might attenuate the beneficial effects of selenium intake on stroke. This may be related to the critical role of erythrocytes in selenium transport [28,29]. The significantly reduced erythrocytes in anaemic patients are insufficient for the transport and utilisation of selenium, which affects the physiological role of selenium.

There is conflicting evidence regarding the association between selenium intake and diabetes. Early case-control studies have shown that diabetes influences serum selenium levels as decreased selenium levels were observed in patients with diabetes [30]. In contrast, observational studies found a positive correlation between serum selenium levels and the incidence of diabetes [30,31]. Secondary analyses in a randomised clinical trial reported that selenium supplementation (200 μg/day) increases the incidence of diabetes [31]. However, other trials did not find an overall significant effect of selenium supplementation (200 μg/day) on incident diabetes [32,33]. Similar contradictions have been reported in relevant systematic reviews and meta-analyses [34,35]. We observed a beneficial effect of dietary selenium intake on the risk of stroke in non-diabetic individuals but not in those with diabetes. It should be noted that the odds ratios of stroke in the two groups were very close [0.72 (0.53, 0.97) for non-diabetes vs. 0.77 (0.53, 1.13) for diabetes]. Therefore, the impact of selenium intake on stroke in diabetes is not yet clear. Further prospective studies are needed.

## Limitation

As a cross-sectional study, stroke history was self-reported, and bias cannot be avoided for some of the results. However, this is likely limited as a previous study confirmed the validity of using self-reported illness to measure objective health [36]. Furthermore, although many covariates were adjusted in the regression models, unmeasured confounders correlated with a stroke cannot be excluded. We can only show correlation, not causality. The impact of selenium on stroke and its specific manifestations in the general population requires further prospective studies.

## Conclusions

In conclusion, Dietary selenium had a negative and non-linear correlation with the risk of stroke in adults. Non-linear negative correlation trends were observed in subpopulations of females, age <60 years, PIR <2.14, overweight and obesity, hypertension, non-diabetes, and non-anaemia. Dietary selenium intake of approximately 105 μg/day has an optimum beneficial effect on stroke.

## Data Availability

The data used in this study are openly available from the Centres for Disease Control and Prevention at https://www.cdc.gov/nchs/nhanes/index.htm
